# Resorcinol Functionalized Gold Nanoparticles for Formaldehyde Colorimetric Detection

**DOI:** 10.3390/nano9020302

**Published:** 2019-02-22

**Authors:** Carlos Martínez-Aquino, Ana M. Costero, Salvador Gil, Pablo Gaviña

**Affiliations:** 1 Instituto Interuniversitario de Investigación de Reconocimiento Molecular y Desarrollo Tecnológico (IDM), Universitat Politécnica de València, Universitat de València, Doctor Moliner 50, Burjassot, 46100 Valencia, Spain; carlos.martinez-aquino@uv.es (C.M.-A.); ana.costero@uv.es (A.M.C.); salvador.gil@uv.es (S.G.); 2 Departamento de Química Orgánica, Universitat de València, Doctor Moliner 50, Burjassot, 46100 Valencia, Spain; 3 CIBER de Bioingeniería, Biomateriales y Nanomedicina (CIBER-BBN), 28029 Madrid, Spain

**Keywords:** gold nanoparticles, colorimetric detection, formaldehyde, resorcinol

## Abstract

Gold nanoparticles functionalized with resorcinol moieties have been prepared and used for detecting formaldehyde both in solution and gas phases. The detection mechanism is based on the color change of the probe upon the aggregation of the nanoparticles induced by the polymerization of the resorcinol moieties in the presence of formaldehyde. A limit of detection of 0.5 ppm in solution has been determined. The probe can be deployed for the detection of formaldehyde emissions from composite wood boards.

## 1. Introduction

Formaldehyde is a pollutant gas emitted from different building materials and domestic products. It is a colorless compound with a strong smell that is widely used in the fabrication of pressed-wood boards, certain insulating materials, and paper product coatings. It is also employed in the preparation of adhesives and glues. Due to its antiseptic properties, its aqueous solution that is called *formalin*, is used as disinfectant not only in different industries but also in hospitals and funeral homes. Other industries such as these related with cosmetics, foods, or medicines also use formaldehyde as a preservative. Materials containing formaldehyde can release formaldehyde gas into the air and for this reason formaldehyde is normally present in both indoor and outdoor air at low levels (40 ppb indoors and 15 ppb outdoors) [1]. Formaldehyde is also an endogenous reactive carbonyl species that is produced through diverse biological processes, such as demethylation of *N*-methylated amino acid and DNA and RNA bases [2].

Some people may experience adverse effects, such as nose and throat irritation, burning sensations in the eyes, or watery eyes when the concentration of formaldehyde present in the air is among 0.5 and 1 ppm [3]. While the immediate effects of exposure to formaldehyde are well established, information on their long-term health effects is more limited. However, the International Agency for Research on Cancer (IARC) includes this compound among the human carcinogens [4]. In the same sense, the National Toxicology Program depending on the Department of Health and Human Services, in 2011, in its *12th Report on Carcinogens* included formaldehyde on the list of dangerous compounds [5]. Due to the widespread use of formaldehyde, its toxicity, and volatility, the development of reliable sensors for its detection is a very active research field.

Detection of formaldehyde in air samples is usually carried out using spectrometric methods [6]. However, high-performance liquid chromatography is preferred when formaldehyde is detected in aqueous media [7,8]. There are alternative methods, such as infrared detection, gas chromatography (GC), polarography, colorimetry, fluorimetry, flow injection analysis, or even gas detector tubes [6,9,10]. Very sensitive techniques that have been also used are HPLC or GC coupled with mass spectrometry [11,12]. However, all these techniques are generally expensive, complex, and require the presence of skilled personnel. In contrast, analyte detection using fluoro- or chromogenic sensors or probes present several advantages. Probes can be used in situ and generally without any sample pre-treatment, they involve low-cost equipment, and, in some cases, the detection can be observed by the naked eye [13]. Although several colorimetric and fluorometric chemosensors for detecting formaldehyde both in solution [14,15,16] and in gas phases [17,18,19] can be found in the literature, new approaches are continuously reported [20].

Among the transduction mechanisms used in the design of colorimetric sensors and probes, the modification in the optoelectronic properties of appropriately functionalized gold nanoparticles (AuNP) has been widely used recently [21]. The sensing protocol is usually based on the analyte-induced aggregation or dispersion of AuNP that modify the surface plasmon band with the corresponding color change. Thus, red colored dispersed nanoparticles become blue upon aggregation and this color change is visible to the eye even at low concentrations [22]. Even though gold and silver nanoparticles have been widely used in sensor design [23], there are very few examples in which these materials have been applied to formaldehyde detection. Ma et al. described the use of surface-enhanced Raman spectroscopy (SERS) for detecting formaldehyde in water and food samples [24]. The sensor works using AgNP and after formaldehyde derivatization with 4-amino-5-hydrazino-3-mercapto-1,2,4-triazole. Wen et al. reported the use of gold nanorods and the dye 4-amino-3-hydrazino-5-mercap-1,2,4-triazole to determine formaldehyde through a resonance Rayleigh scattering-energy transfer process [25]. Fauzia et al. published a biosensor based on AuNP to enhance the response of an appropriate dye. The selectivity was achieved using an alcohol oxidase enzyme (AOX) [26].

**Figure 1:**
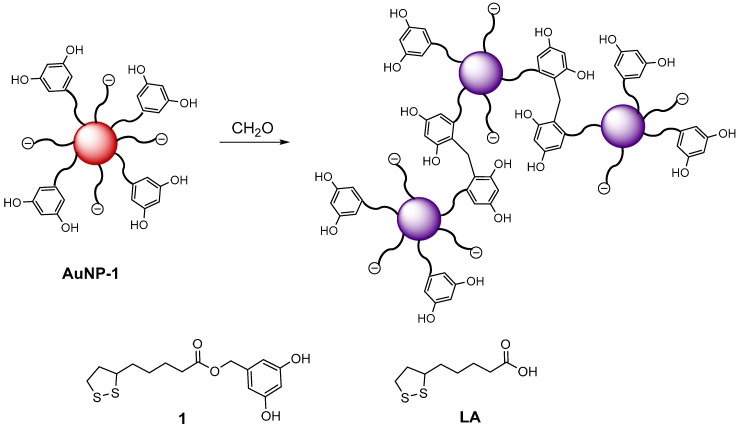
Proposed sensing mechanism and structure of the ligands used in **AuNP-1** preparation. Reaction of the terminal resorcinol with formaldehyde induces cross-linking aggregation of the gold nanoparticles. AuNP: gold nanoparticles; LA: lipoic acid.

On the other hand, it is well known that formaldehyde reacts readily with resorcinol under a mild acid or base catalysis, leading to 3-D polymeric networks through a polycondensation reaction [27]. Considering this fact and our experience in the use of surface modified AuNP in colorimetric sensing applications [28,29], we now report a colorimetric probe for formaldehyde based on gold nanoparticles functionalized with resorcinol. The sensing principle, which is presented in Figure 1, involves the condensation of two resorcinol moieties of different nanoparticles with formaldehyde [30]. This results in interparticle-crosslinking aggregation inducing a change in the surface plasmon resonance (SPR) absorption with the concomitant change in color [31].

## 2. Materials and Methods

### 2.1. General Procedures

Chloroauric acid (HAuCl_4_·3H_2_O), sodium citrate dihydrate, α-lipoic acid, 3,5-dihydroxybenzyl alcohol, diisopropyl azodicarboxylate (DIAD), and triphenylphosphine (TPP) were commercially available and were used without purification. Anhydrous tetrahydrofuran (THF) was freshly distilled from sodium/benzophenone under argon. All the aqueous solutions were prepared with Milli-Q water (18.2 MΩ cm^−1^). A Bruker DPX300 300 MHz spectrometer (Billerica, MA, USA) was used to obtain the ^1^H-NMR spectrum, using tetramethylsilane as internal standard. UV-vis absorption spectra were performed on a Shimadzu UV-2101PC spectrophotometer (Kyoto, Japan). Fourier-transform infrared spectra (FT-IR) were recorded with an Agilent Cary 630 FT-IR spectrometer (Santa Clara, CA, USA). Transmission electron microscopy images were obtained with a JEOL JEM-1010 transmission electron microscope (Tokyo, Japan,) operating at 100 kV. A Malvern Instruments Zetasizer ZS was used for dynamic light scattering (DLS) measurements (Worcestershire, UK, 3 times in 10–25 cycles).

### 2.2. Synthesis of Compound 1

In this study, 3,5-dihydroxybenzyl alcohol (200 mg, 1.40 mmol) was dissolved in dry THF (20 mL). Then, TPP (374 mg, 1 eq.) and DIAD (280 µL, 1 eq.) were added, and the mixture was stirred at room temperature for 5 min under argon. Lipoic acid (294 mg, 1.40 mmol) in dry THF (5 mL) was then added and the mixture stirred at room temperature for 12 h. Solvent was evaporated, and the resulting crude was purified by column chromatography (hexane/AcOEt, 1:1) yielding **1** as a pale ochre solid (14%). ^1^H NMR (300 MHz, CD_3_OD): δ = 1.41–1.53 (m, 2H), 1.59–1.75 (m, 4H), 1.80–1.94 (m, 1H), 2.38–2.51 (m, 3H), 3.03–3.22 (m, 2H), 3.52 (m, 1H), 4.96 (s, 2H), 6.21 (t, *J* = 2.2 Hz, 1H), and 6.28 (d, *J* = 2.2 Hz. 2H) ppm. IR: 3350 (OH), 2950 (Ar–H), 1710 (C=O), 1260 (C–O) cm^−1^.

### 2.3. Synthesis of Probe AuNP-1

All reactions were performed with thoroughly cleaned glassware using aqua regia. In a 250 mL flask, an aqueous solution of HAuCl_4_ (100 mL, 1 mM) was heated to reflux. Then, aqueous trisodium citrate (10 mL, 38.8 mM) was added and the resulting solution was boiled for ca. 30 min until the color of the solution turned red, indicating the formation of citrate-capped AuNPs. Finally, the solution was cooled to room temperature.

To 5 mL of the freshly prepared citrate-capped AuNP, 45 µL of aqueous NaOH 0.5 M was added. Then, resorcinol derivative **1** (50 µL, 0.01 M in MeOH) and lipoic acid (LA) (150 µL, 0.01 M in MeOH) were simultaneously added, and the solution was left at room temperature, with stirring overnight. Finally, the suspension was centrifuged (10,500 rpm, 18 min), the supernatants decanted, and the resulting gold nanoparticles were resuspended in water and stored at 4 °C until its use.

## 3. Results and Discussion

The structure of the resorcinol-terminated ligand **1** used for the functionalization of the gold nanoparticles is shown in Figure 1. Compound **1** was obtained by chemoselective esterification of 5-(hydroxymethyl)resorcinol with lipoic acid (LA) using TPP and DIAD as coupling agents [32].

Citrate-stabilized gold nanoparticles were prepared following the Turkevich method by reducing tetrachloroauric acid with trisodium citrate in boiling water [33]. In a second step, the citrate was displaced from the surface of the nanoparticles by a ligand-exchange reaction with a mixture of LA (to stabilize the nanoparticles suspension in water) and **1** to yield **AuNP-1**. An optimized 1:3 (**1**/LA) molar ratio was used in order to improve the stabilization of the nanoparticles as well as the response towards the analyte, as shown in Figure S1.

**Figure 2:**
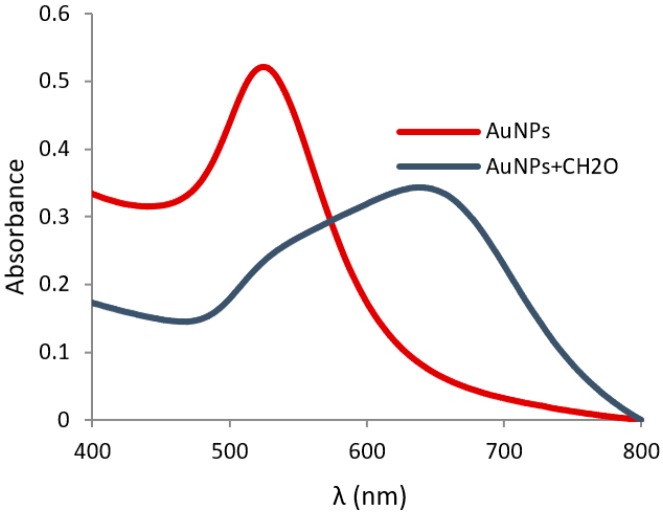
Absorption spectrum of the **AuNP-1** in water at room temperature, in the presence and in the absence of excess (50 mM) formaldehyde.

**Figure 3:**
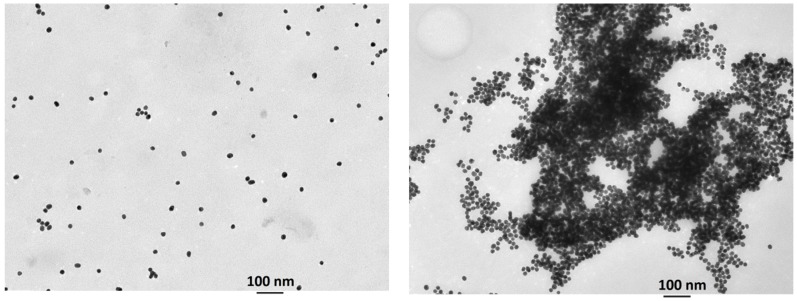
TEM images of stabilized **AuNP-1** dispersion (**left**) and their aggregates upon addition of excess of H_2_CO (**right**).

UV-vis and IR spectroscopy, transmission electron microscopy (TEM), and dynamic light scattering (DLS) were used to characterize the resulting functionalized gold nanoparticles. The aqueous red suspension of **AuNP-1** showed the typical SPR band in the UV-vis spectrum at 525 nm (Figure 2) and the IR spectrum showed bands at 3340, 1728, and 1600–1400 cm^−1^ corresponding to the hydroxyl groups, carbonyl bond, and aromatic moiety respectively, as shown in Figure S2. TEM images showed the presence of monodisperse nanoparticles with an average size of 17 nm, as shown in Figure 3, left. An initial **AuNP-1** concentration of 2.56 × 10^−9^ M was calculated by UV-vis spectroscopy considering a molar extinction coefficient *ε* = 2.5 × 10^8^ M^−1^cm^−1^ [34].

In order to verify the colorimetric response of the probe in the presence of formaldehyde, aqueous suspensions of **AuNP-1** at room temperature were studied by UV-vis spectroscopy, in the absence and in the presence of excess of aqueous H_2_CO. In the absence of the analyte, a stable red wine-colored dispersion was observed, which exhibited the typical SPR absorption band at 525 nm. However, when 50 mM of formaldehyde was added to the probe, after an induction period of ca. 12–15 min, the solution color changed from red to dark blue, and the corresponding UV-vis spectrum showed the appearance of a new SPR absorption peak at 645 nm, characteristic of aggregated gold nanoparticles, as shown in Figure 2.

The observed results agree with the expected condensation of resorcinol moieties on the surface of the gold nanoparticles with formaldehyde. This aggregation process was also confirmed by TEM, as shown in Figure 3, and by DLS measurements, which showed an increase in the hydrodynamic diameter of the nanoparticles from 17 nm to 525 nm in the presence of excess formaldehyde, as shown in Figure S3. Control experiments carried out with citrate-stabilized gold nanoparticles under the same conditions showed no response in the presence of formaldehyde, as shown in Figure S4.

The effect of pH in the sensitivity of the probe towards formaldehyde was evaluated by the UV-vis experiments. Thus, aqueous dispersions of **AuNP-1** nanoparticles were centrifuged, redissolved in different buffers (phosphate, Tris-HCl, and carbonate) at different pH values (from 5.7 to 10) and tested against H_2_CO (10 mM) for 10 min at room temperature. Remarkably, much higher response of the probe in the presence of formaldehyde was observed when the buffer was Tris-HCl at neutral pH, as shown in Figure S5. Thus, all the sensing experiments were performed in 0.01 M Tris-HCl buffer at pH = 7.0. In this medium, the suspensions remained stable for more than two weeks at room temperature, and no obvious change in the characteristic peak at 525 nm was observed, even though the zeta-potential value for the nanoparticles (ca. −22 mV) was not too low as compared to the phosphate buffer (−27 mV), as shown in Figures S6 and S7. By contrast, when phosphate buffered solutions of pHs ranging from 5.7 to 8.0 were used, a much lower response versus formaldehyde was observed in all cases, and the influence of the pH in the response was very small in the pH range studied. These results suggest a catalytic effect of Tris, probably through the formation of an imine intermediate. Finally, a better sensitivity was observed when the experiments were performed at 38 °C after an induction period of 15 min.

**Figure 4:**
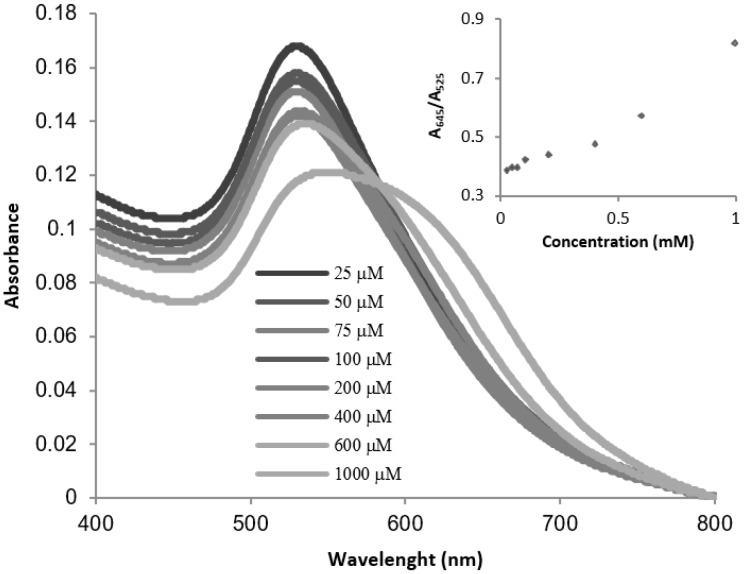
UV-vis absorption spectra of **AuNP-1** (10^−9^ M, in Tris-HCl 0.01 M, pH 7) in the presence of increasing amounts of aqueous formaldehyde. Inset: Plot of the absorption ratio (A_645_/A_525_) versus aqueous formaldehyde concentration.

In order to evaluate the response of the probe in front of formaldehyde and determine its limit of detection (LoD), UV-vis titration studies were performed at 38 °C with buffered aqueous suspensions (pH = 7) of **AuNP-1** and increasing amounts of aqueous H_2_CO, as shown in Figure 4.

**Figure 5:**
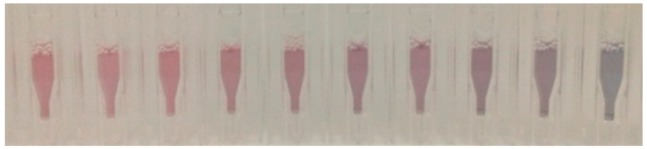
Color changes of AuNPs-1 suspension (Tris-HCl 0.01 M, pH 7) in the presence of increasing amounts of formaldehyde (from left to right, 0 µM, 25 µM, 50 µM, 75 µM, 100 µM, 200 µM, 400 µM, 600 µM, 1 mM, 10 mM).

Under these conditions, a gradual decrease in the SPR band at 525 nm (A_525_) together with an increase in the band at 645 nm (A_645_) was observed in the UV-vis spectra. As shown in Figure 5, these changes in the UV-vis spectra corresponded to a gradual change in the color of the nanoparticle suspensions from pink to blue. An estimated visual LoD (the minimum H_2_CO concentration required for an observable color change) of ca. 0.4 mM (12 ppm) was established.

On the other hand, the relationship between the absorption band at 645 nm and the absorption band at 525 nm (A_645_/A_525_) shows a lineal dependence on formaldehyde concentration in the 25–1000 µM concentration range, as shown in Figure 3, inset. Using these data, a LoD value of 17 µM (0.5 ppm) was determined from the equation: LoD = 3 S_b1_/S, where S_b1_ is the standard deviation of the blank and S is the slope of the calibration curve (see Supplementary Materials).

**Figure 6:**
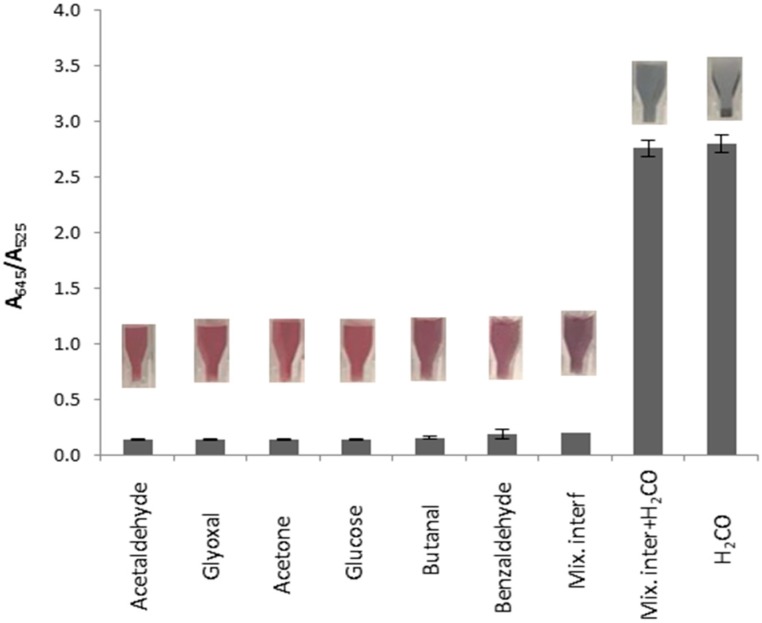
Color changes and UV-vis response of **AuNP-1** in the presence of some possible interferents (100 mM) and a mixture of all and formaldehyde (two replicate experiments were performed).

To evaluate the selectivity of **AuNP-1** towards formaldehyde versus acetone or some common aldehydes, experiments were performed with **AuNP-1** in the presence of acetone, acetaldehyde, glyoxal, butanal, benzaldehyde, or glucose (100 mM each) following the same protocol. As shown in Figure 6, negligible changes in the color or the A_645_/A_525_ ratio were observed in the presence of any of these compounds or in a mixture containing all of them. However, in a competitive experiment in which **AuNP-1** was treated with a mixture containing formaldehyde and all the interferents, the expected color change was observed.

Once we demonstrated the selective response of the probe for aqueous formaldehyde, we decided to extend the probe applicability by detecting formaldehyde gas emission from formalin and from commercial wood boards.

**Figure 7:**
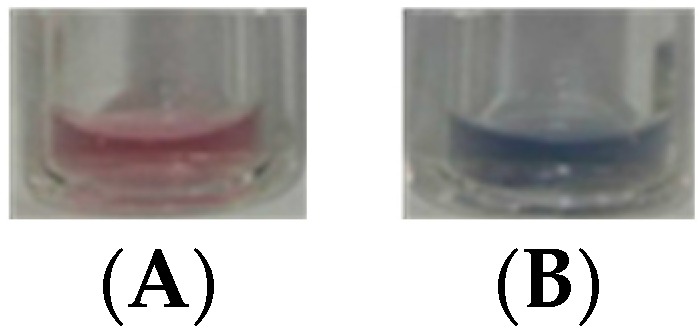
**AuNP-1** suspension (**A**) in absence and (**B**) in presence of gaseous H_2_CO (excess).

Thus, a vial with probe **AuNP-1** was placed inside a closed container, in the presence of ca. 500 ppm of gaseous H_2_CO, which was generated from the liquid-vapor equilibrium of diluted formalin (see Supplementary Materials for details). After 20 min exposure to the gas, a chromogenic change from red to blue was clearly observed in the solution. In a parallel control experiment, a solution of the probe was kept in another container in similar conditions but in the absence of formaldehyde, showing minor color variations throughout the experiment, as shown in Figure 7.

**Figure 8:**
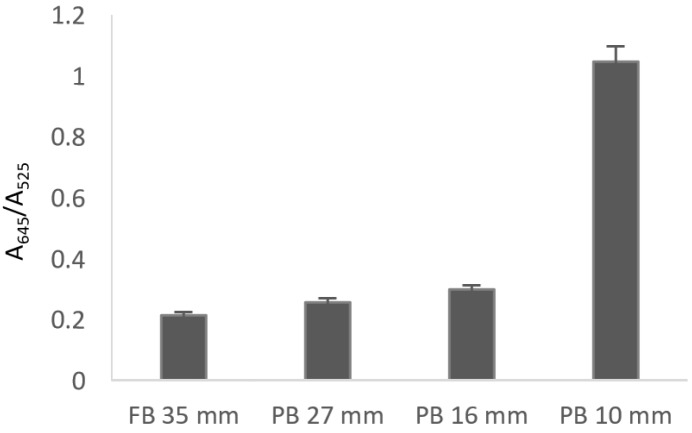
Comparison of formaldehyde emission between fibrous boards (FB) and different particle board (PB) using AuNP-1 (two replicate experiments were performed).

To evaluate the utility of the probe for detecting formaldehyde emission from commercial boards, the corresponding studies were carried out using the previously established conditions. In a typical experiment, board pieces of ca. 15 g were kept close to an open vial containing the probe, all inside of a locked container for 15 hours. After this time, UV spectra of the different probe solutions were registered. Four boards were studied: fiberboard FB 35 mm, particle board PB 27 mm, particle board PB 16 mm, and particle board PB 10 mm, whose formaldehyde content, determined in AIDIMME Technological Institute (Valencia, Spain) by the perforator extraction method, were 5.4, 4.0, 4.5, and 6.2 mg formaldehyde/100 gr dry board, respectively. After recording the UV-vis spectrum of every sample and representing the A_645_/A_525_ ratio for the different kind of fiber and particle boards, the obtained results as shown in Figure 8 indicated that fiberboard showed a lower formaldehyde emission than particleboards having lower formaldehyde content (PB 27 and PB 16 mm). When particleboards with different concentrations of formaldehyde were compared, as expected, the higher content of formaldehyde the higher emission was detected.

## 4. Conclusions

In summary, a simple formaldehyde probe consisting of an aqueous dispersion of resorcinol functionalized gold nanoparticles (AuNP-1) has been synthesized and used for the direct colorimetric detection of formaldehyde both in aqueous media and in gas phases. The detection process is based on a condensation reaction between formaldehyde and the terminal resorcinol moieties of two different nanoparticles. This triggers the aggregation of the dispersed nanoparticles, resulting in a bathochromic shift of the SPR band in the UV-vis spectrum and a change, from red to blue, in the color of the solution, which can be observed by the eye. A remarkable enhancement of the response of the probe towards formaldehyde was observed in the presence of Tris-HCl. Probe AuNP-1 was able to detect formaldehyde in aqueous solution with a limit of detection of 0.5 ppm. The probe was also able to detect gaseous formaldehyde emitted from fiber and particleboard, giving a higher response for the particleboards with higher formaldehyde content.

## Supplementary Materials

The Supplementary Materials are available online at https://www.mdpi.com/2079-4991/9/2/302/s1.
