# Supplementary material for: Resorcinol Functionalized Gold Nanoparticles for Formaldehyde Colorimetric Detection
Source: Nanomaterials (Basel). 2019 Feb 22;9(2):302. doi: 10.3390/nano9020302 (PMC6409679; doi:10.3390/nano9020302)
Supplement: Supplementary file 1 [file nanomaterials-09-00302-s001.pdf]

Resorcinol functionalized gold nanoparticles for formaldehyde colorimetric detection

Carlos Martínez-Aquino[1], Ana M. Costero[1,2,3], Salvador Gil[1,2,3], Pablo Gaviña[1,2,3,*]

[1] Instituto Interuniversitario de Investigación de Reconocimiento Molecular y Desarrollo Tecnológico (IDM). Universitat Politécnica de Valéncia, Universitat de València, Doctor Moliner 50, Burjassot, 46100, Valencia, Spain.
[2] Departamento de Química Orgánica, Universitat de València, Doctor Moliner 50, Burjassot, 46100, Valencia, Spain.
[3] CIBER de Bioingeniería, Biomateriales y Nanomedicina (CIBER-BBN) (Spain).

Optimization of the 1/LA ratio for the synthesis of AuNP-1

These experiments were carried out using the same protocol in all cases: A mixture of functionalized gold nanoparticles (100 µL) and H_2CO (100 µL, 20 mM) was stirred 10 minutes. Then, the absorbance of the different solutions was recorded. The figure S1 shows the ratio between the absorbance at 645 nm and the absorbance at 525 nm (A_{645}/A_{525}) for different **1**/LA concentration ratios.

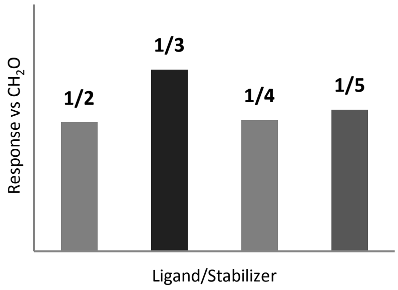

Figure S1. Response of the probe with different ligand/LA ratio versus formaldehyde.

FT-IR spectrum of AuNPs

3 µL of **AuNPs** solution were placed on the IR holder. The sample was air-dried for 1 h and then the spectrum was registered. As can be seen in the **AuNPs-1** spectrum new absorption bands corresponding to carbonyl, aromatic and hydroxyl groups appear. These results confirm the resorcinol functionalization. In the presence of formaldehyde clear changes were observed suggesting the expected polymerization.

(a)

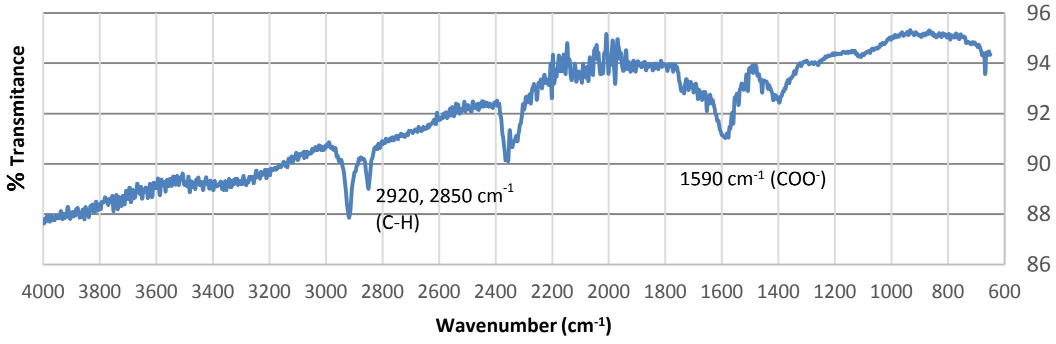

(b)

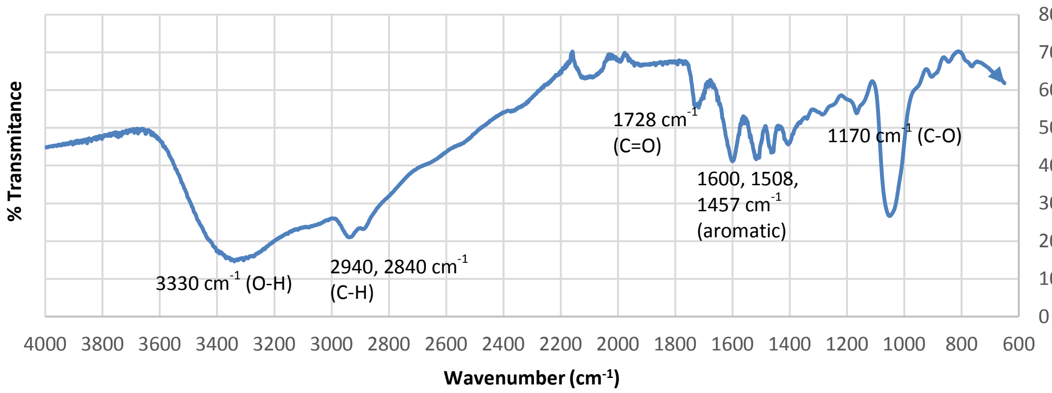

(c)

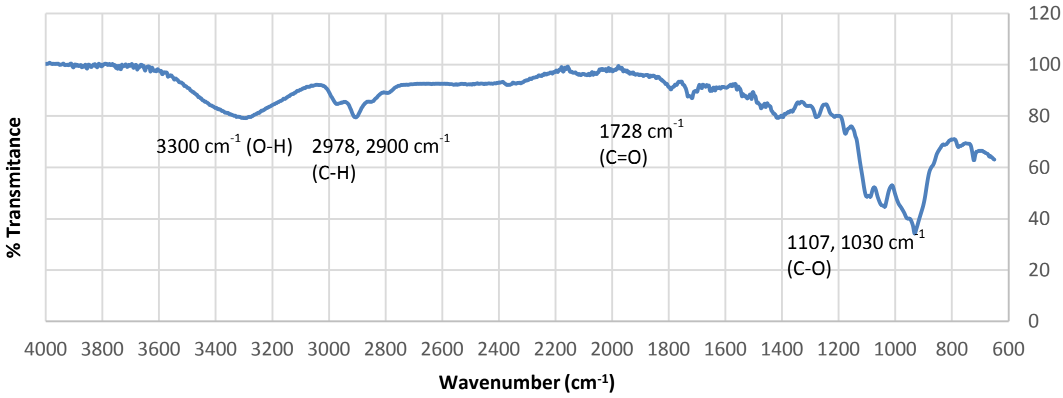

Figure S2. IR spectra of (a) citrate capped AuNPs. (b) **AuNP-1** (c) **AuNP-1** after treatment with formaldehyde.

Dynamic Light Scattering (DLS)

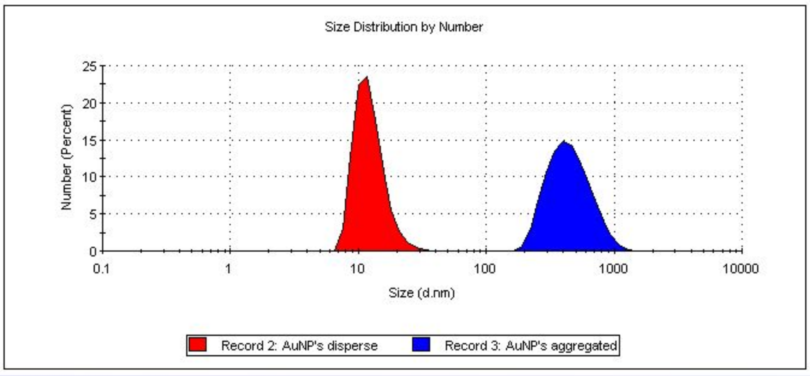

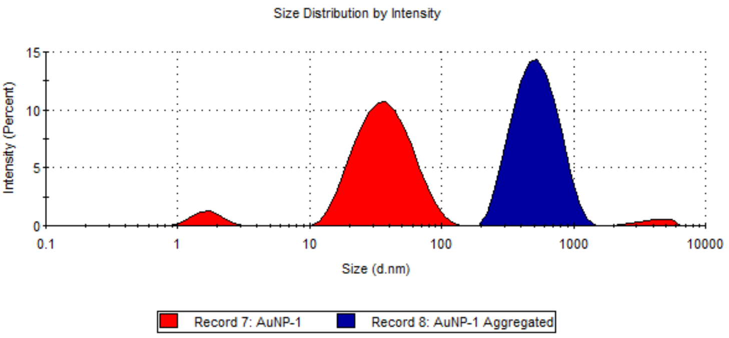

Figure S3. Dynamic light scattering (DLS) measurements of **AuNP-1** in buffered aqueous solution (Tris buffer, pH 7) in the absence (17 nm) (red) and in the presence (525 nm) (blue) of excess of formaldehyde. Top: Size distribution by number (17 nm and 525 nm for dispersed and aggregated respectively). Bottom: Size distribution by intensity (37.8 nm and 531 nm for dispersed and aggregated respectively)

UV-vis spectra of citrate-AuNPs in the absence and presence of formaldehyde

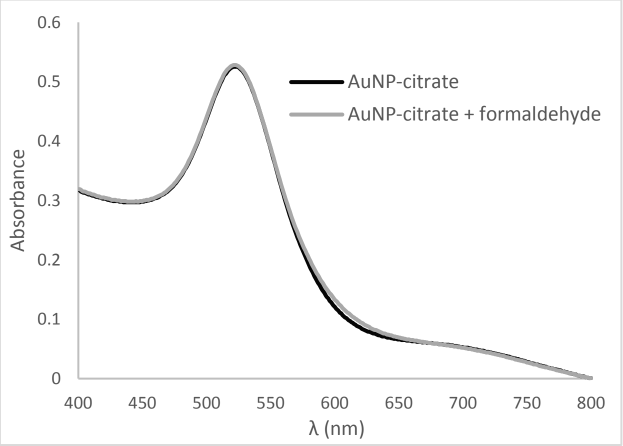

Figure S4. UV-vis spectra of citrate-stabilized gold nanoparticles in water in absence and presence of formaldehyde (50 mM)

Study of the response of the probe to formaldehyde at different pH values

In order to determine best pH and buffer solution for formaldehyde detection, a mixture of **AuNP-1** (100 µL) and H_2CO (100 µL, 20 mM) in distilled water or in different buffered solutions (phosphate, Tris-HCl or carbonate buffers, 10 mM) at different pH values (5.7, 6.0, 6.5, 7.0, 7.5, 8.0 and 10) was stirred for 10 min. Then, the corresponding UV-vis spectra were recorded. Figure S6 shows the A_{645}/A_{525} ratios with the different buffered solutions.

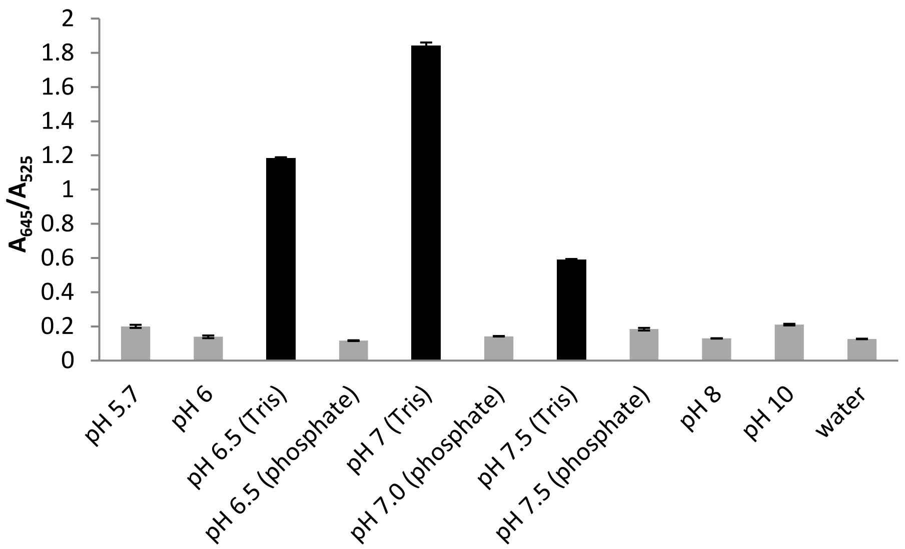

Figure S5. Response of **AuNP-1** versus formaldehyde (10 mM) with different buffered solutions and pH values (Phosphate buffer for pH 5.7, 6.0, 6.5, 7.0, 7.5 and 8; tris-HCl buffer for pH 6.5, 7.0 and 7.5, carbonate buffer for pH 10) (2 replicate experiments were performed).

Zeta-potential studies

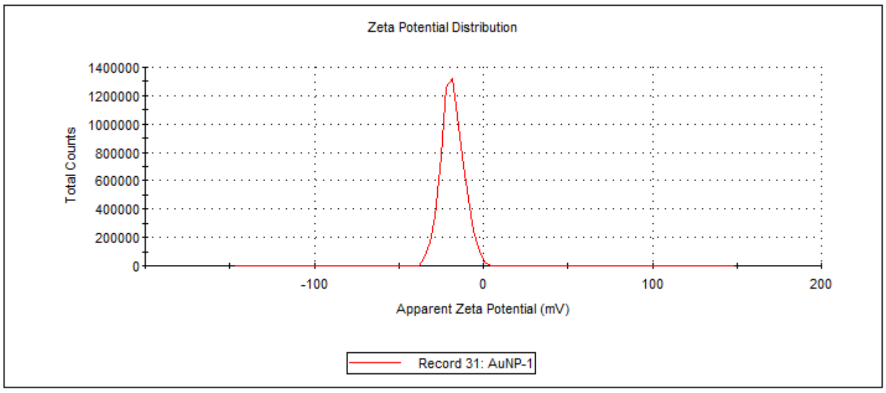

Figure S6. Zeta-potential measurements of **AuNP-1** in buffered aqueous solution (Tis-HCl, pH 7) resulted in -22 mV.

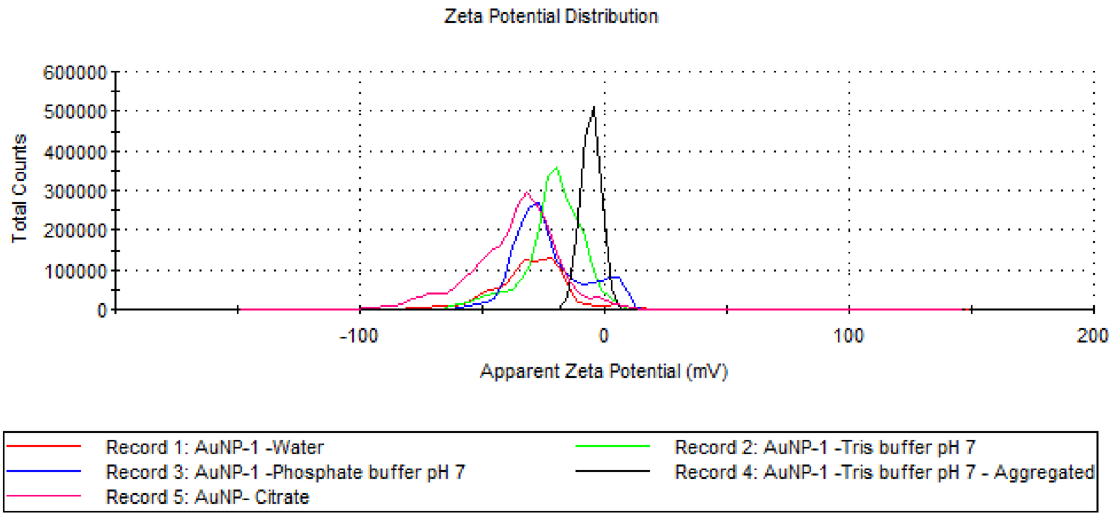

Figure S7. Zeta-potential measurements of **AuNP-1** in water (-21.4 mV), phosphate buffer, pH= 7 (-26.9 mV), Tris-HCl buffer, pH= 7 (-20.0 mV), AuNP-citrate in water (-31.9 mV) and aggregated AuNP-1 in Tris-HCl buffer, pH= 7 (-4.3 mV)

Full UV-vis spectra of citrate-AuNP and AuNP1

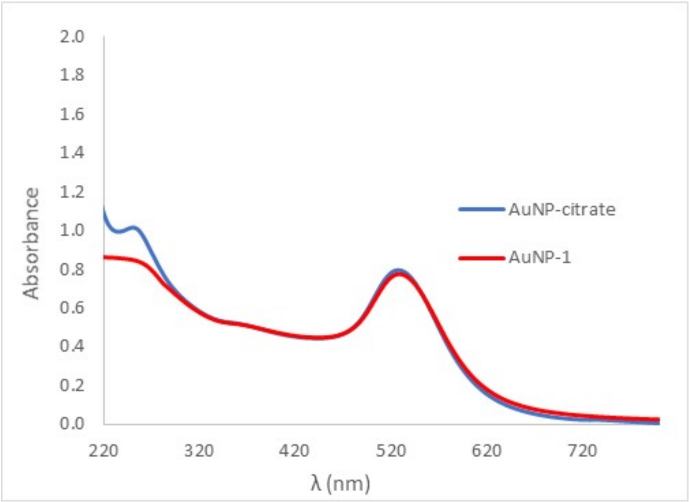

Figure S8. UV-vis spectra of citrate-capped AuNPs in water and **AuNP-1** in Tris-HCl buffer, pH 7.

Titration studies in solution and calculation of limit of detection

Formaldehyde concentration from commercial formalin was determined to be 35% by using the sodium sulfite method. For the titration studies, 100 µL of formaldehyde (35 % or diluted solutions) were added to 100 µL of probe (10^{-9} M, buffered with tris-HCl 10 mM pH 7), reaching

a final formaldehyde concentration between 25 and 1000 µM. The solution was placed in a heater at 38 ºC for 15 min, after that, 200 µL of water was added and the corresponding absorption spectrum was recorded.

In order to evaluate the limit of detection (LoD), UV-vis titration studies were performed with **AuNP-1** and increasing amounts of H_2CO. The absorption band at 525 nm (A_{525}) corresponding to red dispersed AuNPs gradually suffers a batochromic displacement to 645 nm (A_{645}), finally resulting in a blue colour of the solution which can be observed by the naked eye. The visual limit of detection (visual LOD, defined as the minimum concentration of H_2CO necessary for an observable colour change) was 200 µM. LoD was calculated using the equation (1), where K=3, S_b is the standard deviation of the blank (0.0024) and m is the slope of the calibration curve. A LoD value of 17 µM was obtained.

$$\boldsymbol{LoD = K \cdot \frac{Sb}{m}} \qquad (1)$$

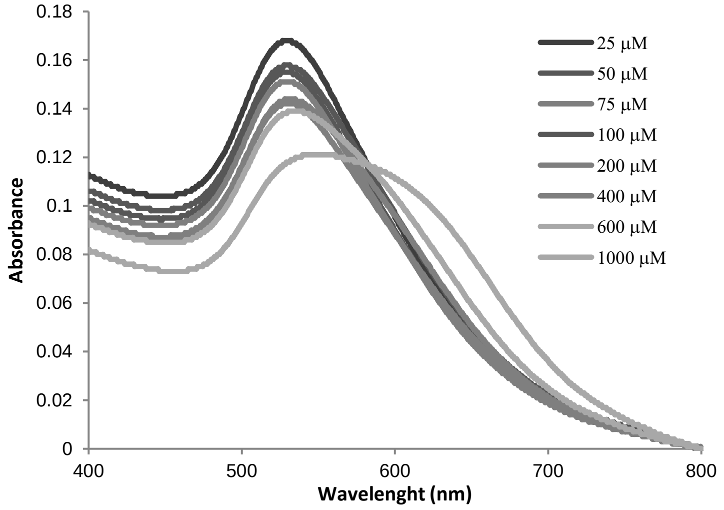

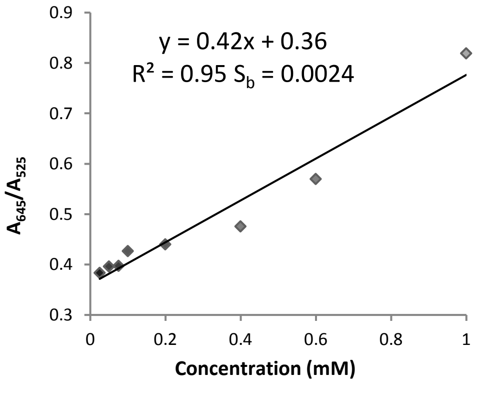

Figure S9. (Top) Plot of absorption spectrum of the probe in presence of increasing amounts of formaldehyde. (Bottom) Plot of the absorption ratio (A_{645}/A_{525}) versus formaldehyde concentration.

Interference studies

For the interference studies, the **AuNPs-1** were exposed to acetaldehyde, glyoxal, acetone, glucose, butanal, benzaldehyde, formaldehyde and a mixture of them. In each case, 100 µl of the interferent (100 mM) was added to 100 µl of **AuNPs-1,** the mixture was stirring for 15 minutes and after that, 200 µL of water was added and the corresponding absorption spectrum was recorded.

Studies in gas phase

A vial containing the probe (400 µL) was placed in a closed container holding the formaldehyde gas generated from the liquid-vapour equilibrium of formalin diluted solutions (5 mL) at 25 ºC, using Henry's law constant to determine the vapour concentrations, and H_{eff} = 3700 M atm^{-1} [1]

$$P(atm) = \frac{Concentration(M)}{H_{eff}(M \cdot atm^{-1})} \quad (2)$$

$$ppm = P(atm) \cdot 10^6 \quad (3)$$

Studies with board pieces

Fiberboard (FB 35 mm), and particleboards (PB 27 mm, PB 16 mm and PB 10 mm), whose formaldehyde content, determined by the perforator extraction method, were 5.4, 4.0, 4.5 and 6.2 mg formaldehyde/100 gr dry board respectively, were provided by the Valencia Technological Institute AIDIMME.
In a typical experiment, board pieces (squares with a surface of 2.5 cm x 2.5 cm, 15 g approximately) were placed next to an open vial containing the probe (300 µL of **AuNPs-1**, 9.72 x 10^{-10} M) inside of a locked container for 15 hours. After this time, UV spectra of the different probe solutions were registered.

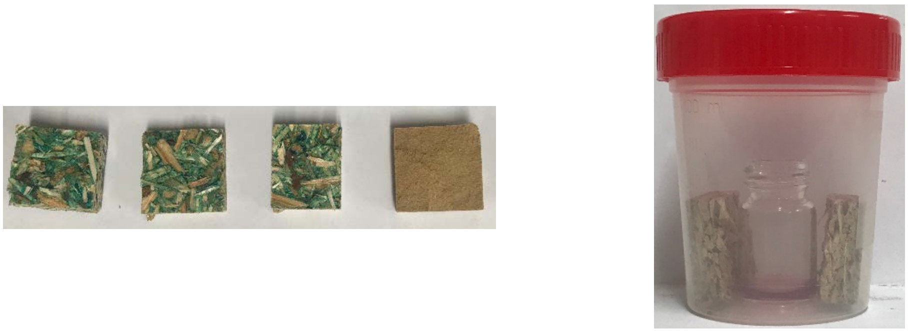

Figure S10. Left (different board pieces, from left to right PB 27 mm, PB 10 mm, PB 16 mm, FB 35 mm). Right (system for the detection of the formaldehyde emission from board pieces).

References

[1] Liu, X.; Guo, Z.; Roache, N. F.; Mocka, C. A.; Allen, M. R.; Mason, M. A. *Environ. Sci. Technol.*, **2015**, *49*, 1603.
